# The Clinical and MRI Effect of TNF-α Inhibitors in Spondyloarthritis Patients With Hip Involvement: A Real-World Observational Clinical Study

**DOI:** 10.3389/fimmu.2021.740980

**Published:** 2021-09-29

**Authors:** Kui Zhang, Yan Zheng, Qing Han, Ying Liu, Weitao Wang, Jin Ding, Yan Wang, Bei Zhang, Junfeng Jia, Minwen Zheng, Zhaohui Zheng, Ping Zhu

**Affiliations:** ^1^ Department of Clinical Immunology, Xijing Hospital, The Fourth Military Medical University, Xi’an, China; ^2^ National Translational Science Center for Molecular Medicine, Xi’an, China; ^3^ Department of Radiology, Xijing Hospital, The Fourth Military Medical University, Xi’an, China

**Keywords:** MRI, hip, spondyloarthritis, TNF-α, ASDAS-ESR

## Abstract

**Objectives:**

Hip involvement is an important cause of disability and poor prognosis in patients with spondyloarthritis (SpA). Tumor necrosis factor (TNF)-α inhibitor treatment has been demonstrated to be effective in SpA patients with hip arthritis; however, quantitative assessment using MRI in long-term follow-up needs further application and observation.

**Methods:**

A total of 239 patients were involved in this study. Methotrexate and sulfasalazine were given as basic treatment. In total, 165 patients received TNF-α inhibitors plus basic treatment, and 74 received basic treatment only, as controls. Clinical symptoms were assessed at baseline and at weeks 12, 24, and 52. MRI performances of hip arthritis, including bone marrow edema (BME) and synovitis, were quantitatively assessed using the Hip Inflammation MRI Scoring System (HIMRISS).

**Results:**

The clinical values of erythrocyte sedimentation rate (ESR), C-reactive protein (CRP), Bath Ankylosing Spondylitis Disease Activity Index (BASDAI), Harris hip score, and Ankylosing Spondylitis Disease Activity Score (ASDAS)-ESR in both groups showed significant clinical remission at week 52 (p < 0.001). However, the change in disease activity levels at week 52 in the control group was significantly worse than in the TNF-α inhibitor group. At week 52, MRI showed a significant remission trend in the TNF-α inhibitor group versus baseline, and total HIMRISS scores were significantly decreased (26.49 ± 10.37 *vs.* 20.59 ± 9.41, p < 0.001); the control group only had slight improvement (p < 0.05).

**Conclusions:**

TNF-α inhibitors could significantly improve clinical and MRI manifestations of hip involvement in patients with SpA. Quantitative MRI assessment combined with clinical assessment can be used to accurately evaluate the treatment effect of TNF-α in SpA patients with hip involvement to help guide targeted treatment.

## Introduction

Hip involvement is an important cause of disability and poor prognosis in approximately 10%–50% of patients with spondyloarthritis (SpA) (1, 2), and 47%–90% of those patients with hip involvement have it bilaterally (3). Progression of hip involvement is reported to be associated with more severe spinal involvement (4, 5) and seriously affects joint function and life quality. In addition, nearly 8% of patients with hip involvement have intractable pain and disability. Even if they are treated with total hip arthroplasty, there is a high likelihood of revision surgery and high rates of complication (6).

The principal clinical manifestations of hip involvement in SpA include inflammation of the subchondral bone marrow edema (BME) and synovitis effusion (7). MRI has been widely used as a sensitive tool to detect hip arthritis. MRI is helpful for early diagnosis to enable treatment to suppress inflammation and avoid further structural damage (8, 9). The Hip Inflammation MRI Scoring System (HIMRISS) is a new quantitative assessment method that is based on several MRI slices from one patient. The HIMRISS is used to systematically assess synovitis and BME of the femoral head and acetabulum (10). Although the association between the HIMRISS and disease activity has been validated in SpA (11), the sensitivity and accuracy of the HIMRISS in assessing treatment response in SpA with hip involvement require further validation.

Previous SpA treatment strategies have focused on global symptom management rather than SpA-related hip lesions or other peripheral joint diseases (12, 13). Increasing evidence indicates that early detection and diagnosis of hip inflammation are beneficial to active treatment, which is important to improve hip function and general prognosis of SpA (14). Tumor necrosis factor (TNF)-α inhibitors are a prompt and robust treatment to improve the signs and symptoms of SpA as well as function and spinal mobility (15, 16). In recent clinical studies, TNF-α inhibitors, including etanercept, adalimumab, and infliximab, were shown to be effective for patients with SpA patients with hip involvement in clinical and imaging assessments (1). However, the detailed clinical effects of TNF-α inhibitors in the long-term treatment cycle need to be further clarified. Previous imaging assessments have been limited to radiographic assessment of Bath Ankylosing Spondylitis Radiology Index (BASRI)-hip or ultrasound (17, 18). More accurate imaging tools such as MRI are not sufficiently applied in assessment of the effect of acute and long-term follow-up treatment with TNF-α inhibitors.

Thus, the purpose of this prospective study was to quantitatively assess the details of clinical remission and MRI changes in hip involvement under treatment with TNF-α inhibitors. Hip inflammation was quantitatively assessed using HIMRISS based on MRI changes, as well as systematic clinical evaluation. We aimed to suggest an assessment standard for patients with SpA patients with hip involvement, including MRI and clinical standards, to help guide accurate and targeted treatment.

## Materials and Methods

### Study Population

This clinical trial was registered in the Chinese Clinical Trial Registry on November 28, 2011 (ID: ChiCTR-ONRC-11001846, http://www.chictr.org.cn/showproj.aspx?proj=7701). The study was based on data from Xijing Hospital in China. All patients with a clinical diagnosis of SpA were enrolled, and long-term treatment and monitoring were conducted by rheumatologists in Xijing Hospital. Eligible patients were 18–65 years of age and fulfilled the recently published Assessment of SpondyloArthritis international Society (ASAS) classification criteria (19) or the Modified New York criteria for SpA (20).

The inclusion criteria for hip involvement were subject with i) spontaneous groin, thigh, and hip pain with or without history of trauma; ii) limited internal and external rotation of the symptomatic hip/hips after initial clinical assessment; and iii) both acute and chronic inflammatory changes on MRI.

Patients were excluded from this study if they had i) hip surgery or hip trauma in the previous year; ii) pregnancy; iii) any condition that would limit lower extremity function and mobility, such as history of stroke, infection, lower extremity joint replacement, or amputation; iv) abnormal laboratory test at 4 weeks before treatment (routine blood test: hemoglobin <90.0 g/L, white blood cell count <3.0 × 10^9^/L, and neutrophils <1.5 × 10^9^/L; biochemical test: alanine aminotransferase (ALT) or aspartate aminotransferase (AST) or total bilirubin >normal upper limit, and positive for HIV or syphilis); v) MRI contraindications (e.g., implanted pacemaker or claustrophobia); or vi) if the acquired MRI images were suboptimal in quality.

In order to enhance treatment of SpA patients with hip involvement and reduce disability rate, all enrolled participants would receive basic treatment with methotrexate (10 mg every week) and sulfasalazine (SSZ; 2 g/day, given orally) from baseline to 52 weeks. For all eligible participants, patients who were disinclined to have TNF-α inhibitor or with TNF-α inhibitor contraindications such as the patients with a hepatitis B virus and mycobacterium tuberculosis infection were allocated to the control group and would receive basic treatment only. Other patients were allocated to the TNF-α group and received one of three kinds of TNF-α inhibitor plus basic treatment (etanercept 50 mg given once weekly subcutaneously (s.c.); adalimumab treatment 40 mg s.c. every other week; infliximab 3 mg/kg i.v. at baseline, week 2 and week 6, and then every 8 weeks). The cost of treatment was self-funded, and therapeutic options were determined on the consent of the doctors and patients. The study was approved by the ethics committee of Xijing Hospital, and all patients provided written informed consent before enrollment in this study.

Erythrocyte sedimentation rate (ESR), C-reactive protein (CRP), and human leukocyte antigen B27 (HLA-B27) were measured with standard laboratory techniques. Serum CRP and ESR were measured at baseline and at 12, 24, and 52 weeks. At the same time point, evaluation of disease activity levels (Bath Ankylosing Spondylitis Disease Activity Index (BASDAI), Ankylosing Spondylitis Disease Activity Score (ASDAS)-CRP, and ASDAS-ESR) and hip function level [Harris hip score (HHS)] were conducted by experienced rheumatologists. At the same time point, the ASAS remission rates (including ASAS20, ASAS 40, ASAS50, and ASAS partial) were also calculated for the TNF-α group.

### Quantitative MRI Assessment of Changes

The HIMRISS was used to assess the changes on MRI between baseline and week 52. MRI was performed with a coronal short tau inversion recovery (STIR) sequence with slice thickness of 4 mm and field of view 400 × 400 mm. The baseline and follow-up MRI examinations of both hips were recommended to be performed every 6 months during the 52-week follow-up. And the MRI scans at baseline and at week 52 were calculated by HIMRISS. The HIMRISS protocol was used to score the inflammatory changes on MRI images of the hip joints including BME of the femoral head, BME of the acetabulum, and synovitis (10, 11). The HIMRISS procedure was performed independently by two readers (YL and MZ) who had been well trained at a previous study (11). The final HIMRISS scores were mean scores of both readers. Statistical analysis was conducted by an independent technologist (YZ). The standard HIMRISS scores were calculated based on 15 MRI image slides; therefore, to avoid bias, patients with MRI images of the hip structure who had fewer than five slides were considered as missing samples.

### Statistical Analysis

The Kaplan–Meier test was used to assess the differences in remission of clinical symptoms (ASAS20, ASAS40, ASAS50, and ASAS partial) and MRI improvement between different follow-up time points. The baseline status was taken into account, and non-parametric analysis of covariance was used. The statistical analyses were performed using IBM SPSS 22.0 (IBM Corp., Armonk, NY, USA). p-Values <0.05 were considered to be statistically significant.

## Results

### Demographic and Clinical Characteristics

Between January 1, 2014, and December 30, 2018, we enrolled 239 patients with SpA and coxitis, and treatment follow-up was completed in these patients. In total, 165 patients (69.04%) received TNF-α treatment, and 74 controls (30.96%) received basic treatment only (**Table 1**). In the TNF-α inhibitor group, 103 patients received etanercept treatment, 51 received adalimumab, and 11 patients received infliximab treatment. Patients with SpA in both the TNF-α inhibitor group and control group underwent evaluation of clinical symptoms and MRI examination during the 52-week observation period. As shown in **Table 1**, the differences in demographic and clinical characteristics including ESR, CRP, Harris, BASDAI, morning stiffness time, and ASDAS-CRP between the two groups were not significant at baseline, except for ASAS-ESR (p < 0.01) and disease duration (p < 0.001). Patients in the TNF-α inhibitor group had shorter disease duration and worse disease activity than the control group. All patients had serious disease activity with high BASDAI scores (5.76 ± 1.20 and 5.53 ± 1.29) and ASDAS score (2.78 ± 0.62 and 2.53 ± 0.57), as well as elevated CRP and ESR values.

**Table 1:** Clinical characteristics including disease activity indexes and hip function in both groups at baseline.

	TNF-α group	Control group	p-Value
Number	165	74	
Age	28.67 ± 10.21	27.3 ± 7.11	0.362
Sex	117:48 = 71%:39%	43:31 = 58%:42%	0.067
B27	93%	87%	0.163
Disease duration (m)	25.19 ± 34.52	45.41 ± 40.48	0.000
ESR (mm)	26.91 ± 14.90	25.04 ± 14.34	0.367
CRP (mg/L)	1.42 ± 1.28	1.27 ± 1.23	0.364
Harris	67.65 ± 9.26	66.27 ± 10.85	0.313
BASDAI	5.76 ± 1.20	5.53 ± 1.29	0.199
Morning stiffness (h)	0.43 ± 0.18	0.39 ± 0.42	0.276
ASDAS-ESR	2.76 ± 0.60	2.90 ± 0.67	0.124
ASDAS-CRP	2.78 ± 0.62	2.53 ± 0.57	0.004

ESR, erythrocyte sedimentation rate; CRP, C-reactive protein; BASDAI, Bath Ankylosing Spondylitis Disease Activity Index; ASAS, Assessment of SpondyloArthritis international Society.

### Quantitative MRI Assessment

MRI changes in hip inflammation among patients with SpA were assessed using the HIMRISS, including BME and synovitis. Compared with those at baseline, patients in the TNF-α inhibitor group had significant improvement on MRI of both BME and synovitis at week 52 (all p < 0.001). However, the difference on MRI between week 52 and baseline in the control group was smaller (BME of the femoral head, p < 0.05; total BME, p < 0.05; synovitis, p < 0.05; total mean/max HIMRISS, p < 0.05); BME scores of the acetabulum were similar (p > 0.01) (**Table 2**).

**Table 2:** HIMRISS values from baseline to 52 weeks in the biological group and non-biological group.

	Group	Baseline	Treatment 52 weeks	Changed levels	Baseline *vs.* week 52 p-value
BME of femoral head^mean^ (0–65)	TNF-α group	10.53 ± 6.78	7.74 ± 5.77	2.86 ± 0.38	0.000
	Control group	9.74 ± 5.77	8.73 ± 5.06	0.74 ± 0.40	0.018
	TNF *vs.* control p-value	0.124	0.315	0.001	
BME of acetabular^mean^ (0–35)	TNF-α group	7.06 ± 3.68	5.51 ± 3.81	1.60 ± 0.23	0.000
	Control group	6.27 ± 2.82	5.89 ± 2.45	0.33 ± 0.26	0.164
	TNF *vs.* control p-value	0.006	0.000	0.028	
Total BME^mean^ (0–100)	TNF-α group	17.59 ± 9.36	13.26 ± 8.75	4.46 ± 0.52	0.000
	Control group	16.05 ± 7.11	14.61 ± 2.44	1.06 ± 0.55	0.019
	TNF *vs.* control p-value	0.047	0.037	0.005	
Synovitis effusion score^mean^ (0–30)	TNF-α group	8.90 ± 3.33	7.34 ± 2.97	1.53 ± 0.23	0.000
	Control group	8.44 ± 2.19	7.71 ± 2.24	0.48 ± 0.23	0.011
	TNF *vs.* control p-value	0.002	0.088	0.000	
Total HIMRISS^ave^ (0–130)	TNF-α group	26.49 ± 10.37	20.59 ± 9.41	5.99 ± 0.58	0.000
	Control group	24.49 ± 8.07	22.33 ± 7.07	1.54 ± 0.66	0.039
	TNF *vs.* control p-value	0.073	0.079	0.011	
Total HIMRISS^max^ (0–130)	TNF-α group	29.95 ± 11.70	23.03 ± 10.31	7.07 ± 0.66	0.000
	Control group	27.31 ± 9.50	24.64 ± 8.38	1.91 ± 0.73	0.015
	TNF *vs.* control p-value	0.118	0.126	0.007	

ave, the average score of two hips; max, the max score of two hips; changed levels, score at baseline minus score at week 52; HIMRISS, Hip Inflammation MRI Scoring System; BME, bone marrow edema.

Intergroup analysis showed little difference between the total HIMRISS scores of the TNF group and control group at baseline (p > 0.05) and at week 52 (p > 0.05). However, the treatment group with higher total HIMRISS scores at baseline demonstrated significant improvement at week 52 and lower scores than did the control group (TNF-α *vs.* control groups at week 52: 20.59 ± 9.41 *vs.* 22.33 ± 7.0). Additionally, acetabular BME scores indicated that patients in the TNF-α group had significantly more serious disease activity at baseline (p = 0.006) but had significant improvement at week 52 (p < 0.001) (**Table 2**).

### Clinical Symptoms and Hip Function Assessment

Both the TNF-α inhibitor group and control group achieved significant amelioration of all clinical symptoms and hip function at week 52, but the improvement degrees of TNF group were significantly better than those of the control group (**Table 3**). Notably in TNF-α inhibitor group, BASDAI scores decreased from 5.76 ± 1.20 to 2.16 ± 1.90 at week 52 (p < 0.001); ASDAS-CRP/ASDAS-ESR decreased from 2.78 ± 0.62/2.76 ± 0.60 to 1.06 ± 0.46/1.42 ± 0.60 at week 52 (p < 0.001); CRP and ESR levels also improved significantly compared with baseline levels (p < 0.001 and p < 0.001). In addition, Harris score indicated significant hip function improvement to generally normal by week 52 versus baseline in the TNF-α inhibitor group (67.65 ± 9.26 *vs.* 92.26 ± 7.06, p < 0.001).

**Table 3:** Clinical outcomes and hip function from baseline to 52 weeks between the TNF-α inhibitor group and control group.

	Groups	Baseline	Treatment 52 weeks	p-Value
ESR (mm)	TNF-α group	26.91 ± 14.90	11.01 ± 12.58	0.000
	Control group	25.04 ± 14.34	17.07 ± 12.23	0.000
	**p-Value**	0.367	0.002	
CRP (mg/L)	TNF-α group	1.42 ± 1.28	0.73 ± 1.17	0.000
	Control group	1.27 ± 1.23	0.88 ± 0.91	0.047
	**p-Value**	0.364	0.371	
Harris	TNF-α group	67.65 ± 9.26	92.26 ± 7.06	0.000
	Control group	66.27 ± 10.85	82.95 ± 7.93	0.000
	**p-Value**	0.313	0.000	
BASDAI	TNF-α group	5.76 ± 1.20	2.16 ± 1.90	0.000
	Control group	5.53 ± 1.29	2.81 ± 1.42	0.000
	**p-Value**	0.199	0.000	
ASDAS-CRP	TNF-α group	2.78 ± 0.62	1.06 ± 0.46	0.000
	Control group	2.53 ± 0.57	1.40 ± 0.63	0.000
	**p-Value**	0.004	0.000	
ASDAS-ESR	TNF-α group	2.76 ± 0.60	1.42 ± 0.60	0.000
	Control group	2.90 ± 0.67	1.88 ± 0.62	0.000
	**p-Value**	0.124	0.000	

ESR, erythrocyte sedimentation rate; CRP, C-reactive protein; BASDAI, Bath Ankylosing Spondylitis Disease Activity Index; ASDAS, Ankylosing Spondylitis Disease Activity Score.

Even the control group showed a significant clinical improvement at week 52 versus baseline (p < 0.001); the mean BASDAI score, ASDAS-CRP, ASDAS-ESR, and Harris scores of the TNF-α group were significantly better than those of the control group at week 52 (all p-values <0.001) (**Table 3**). In particular, the degree of ASDAS-CRP indicated a real-world clinical characteristic. Patients in the TNF-α group had more serious disease activity at baseline but obviously better remission at week 52 than had controls.

**Figure 1:**
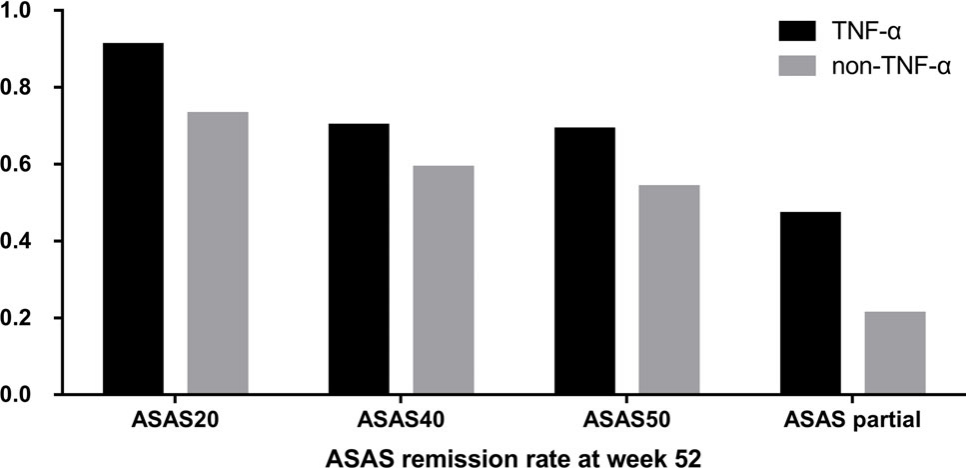
Distribution of Assessment of SpondyloArthritis international Society (ASAS) remission rates between the group that received tumor necrosis factor (TNF)-α inhibitors and the control group at week 52.

**Figure 2:**
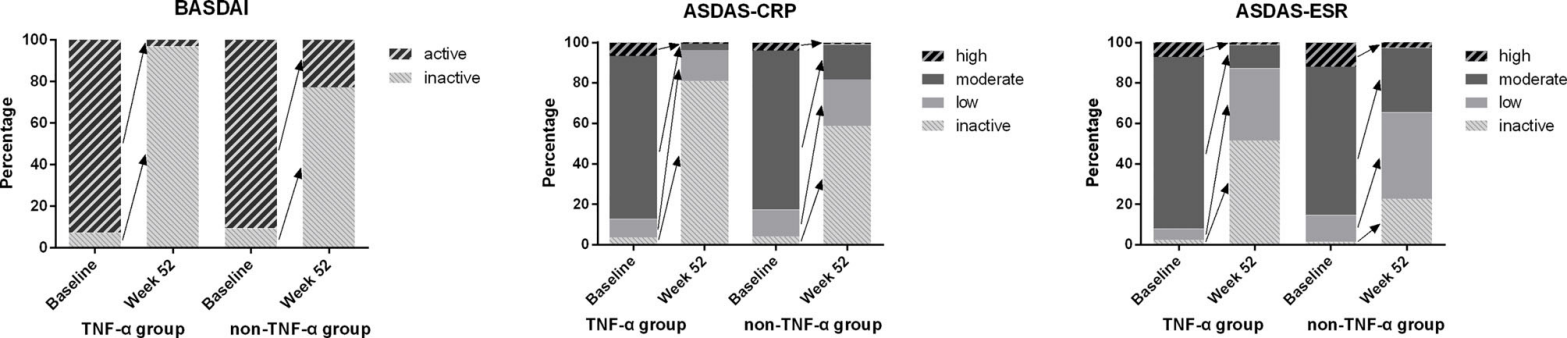
Distribution of disease activity between baseline and week 52 in both groups. Notes. Inactive disease: ASDAS-CRP ≤ 1.3, ASDAS-ESR ≤ 1.3, or BASDAI ≤ 4. Low disease activity: 1.3 < ASDAS-CRP/ASDAS-ESR ≤ 2.1. Moderate disease activity: 2.1 < ASDAS-CRP/ASDAS-ESR ≤ 3.5. High disease activity: ASDAS-CRP/ASDAS-ESR > 3.5. Active disease: BASDAI > 4. ASDAS, Ankylosing Spondylitis Disease Activity Score; CRP, C-reactive protein; ESR, erythrocyte sedimentation rate; BASDAI, Bath Ankylosing Spondylitis Disease Activity Index.

The ASAS remission rates of ASAS20, ASAS40, and ASAS50 and the ASAS partial remission rate were compared between the TNF-α group and control group at week 52. The TNF-α group had higher and better ASAS remission levels than the control group (**Figure 1**). Moreover, the distribution of disease activity including BASDAI, ASDAS-CRP, and ASDAS-ESR between the two groups at week 52 was significantly different. Patients in the TNF-α inhibitor treatment group were able to achieve inactive disease status (TNF-α *vs.* controls: BASDAI, 96.93% *vs.* 74.33%; ASDAS-CRP, 80.98% *vs.* 58.67%; ASDAS-ESR, 51.53% *vs.* 22.67%; p < 0.001). However, a considerable number of patients in the control group were still classified as having active disease (TNF-α *vs.* controls: BASDAI, 3.07% *vs.* 22.67%; p < 0.001), low disease activity (TNF-α *vs.* controls: ASDAS-CRP, 15.34% *vs.* 24.00%; ASDAS-ESR, 35.58% *vs.* 42.67%; p < 0.001), and moderate disease activity (TNF-α *vs.* controls: ASDAS-CRP, 3.07% *vs.* 17.33%; ASDAS-ESR, 11.66% *vs.* 32.00%; p < 0.001) (**Figure 2**).

**Figure 3:**
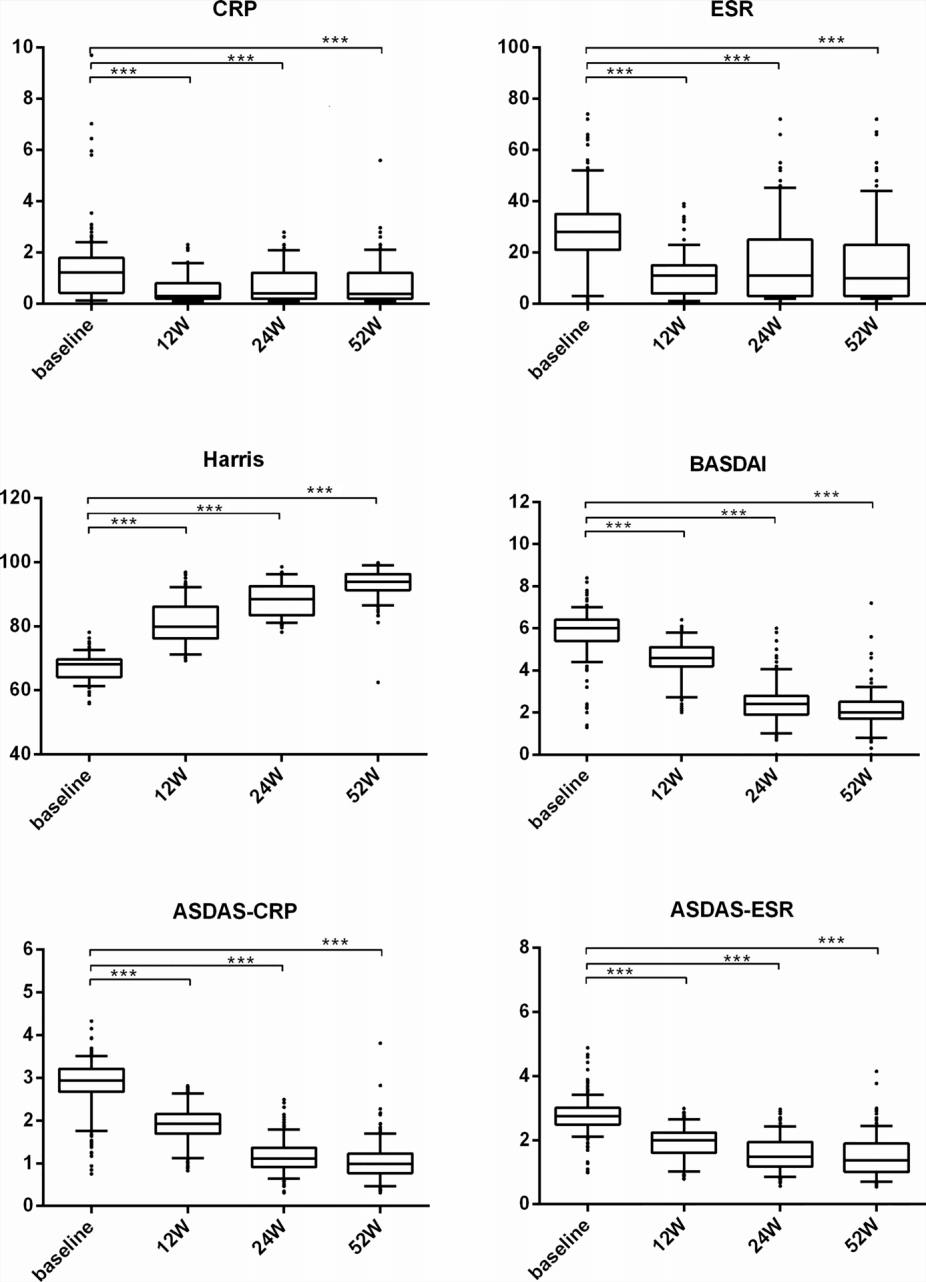
The clinical performance of TNF-α inhibitor group from baseline to week 52. For each box-and-whisker plot, the whisker represents the range, the box represents the 5th–95th percentile, the solid lines within the box represent the mean values of clinical indexes, ***p < 0.001.

**Figure 4:**
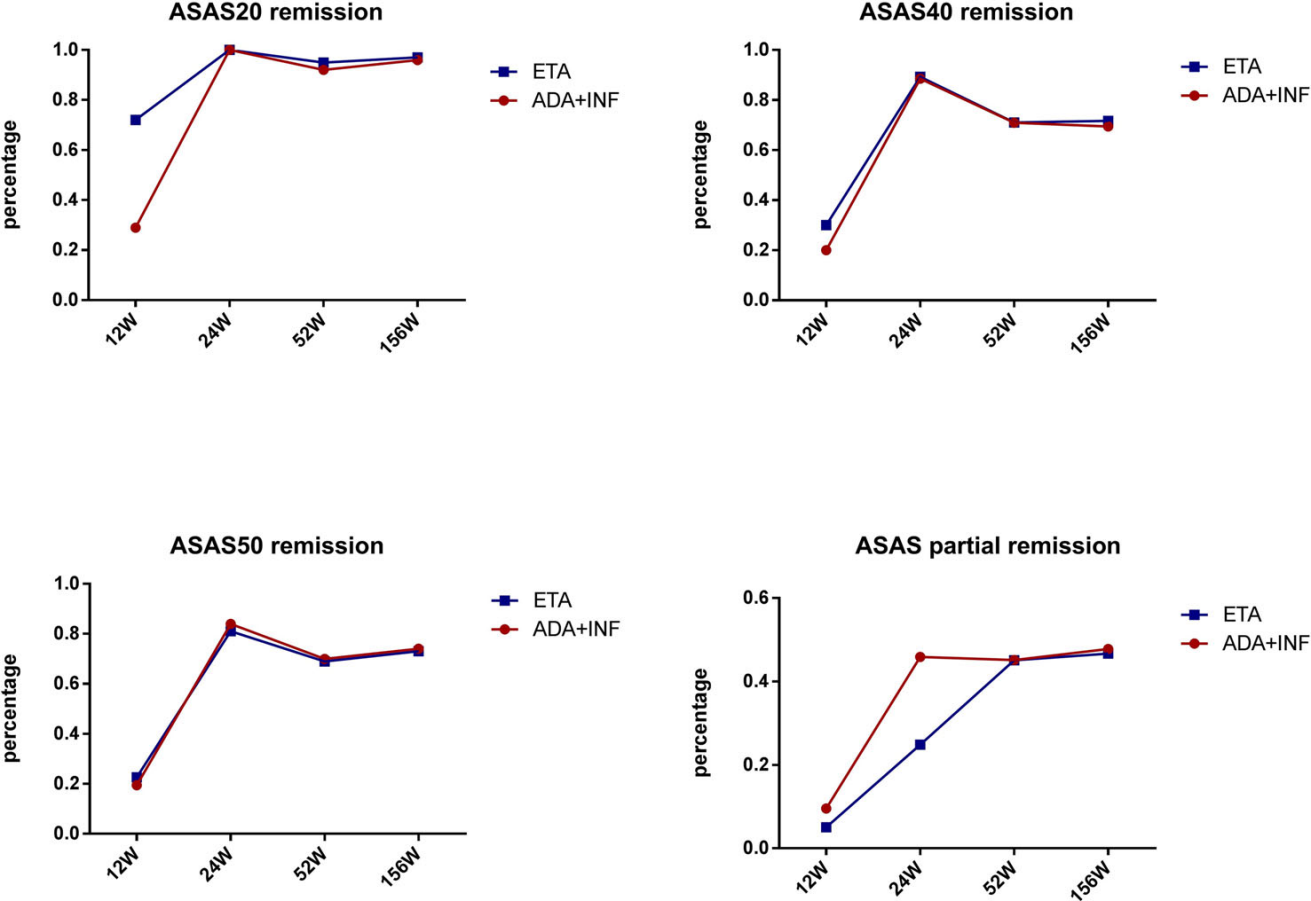
ASAS remission rates between groups receiving different TNF-α inhibitors. ETA, etanercept; ADA, adalimumab; INF, infliximab; ASAS, Assessment of SpondyloArthritis international Society.

We also performed subgroup analysis of all patients in the TNF-α group to compare the treatment difference between different TNF-α inhibitors. First, for all patients receiving treatment with TNF-α inhibitors, all continuous parameters related to disease activity (i.e., CRP, ESR, BASDAI, and ASDAS) and hip joint function (i.e., Harris) were significantly ameliorated from week 12 (all p < 0.001) compared with baseline, and the significant remission levels persisted until week 52 (all p < 0.001) (**Figure 3**). ASAS remission rates, including those of ASAS20, ASAS40, and ASAS50, and ASAS partial remission rates were compared between different TNF-α inhibitors and follow-up times. The results indicated that the differences in ASAS remission rates between different TNF-α inhibitor groups during the 52 weeks were not significant (p > 0.01) (**Figure 4**). Additionally, the HIMRISS was also compared between different TNF-α inhibitor groups (**Supplementary Table S1**). Both TNF-α inhibitor groups had achieved significant imaging improvement at week 52, and the changed HIMRISS levels were similar between different TNF-α inhibitor groups. The treatment responses were similar, which may be consistent with the clinical presentations.

Further, correlation analysis between HIMRISS and clinical outcome was also performed for both groups (**Supplementary Table S2**). In the TNF-α group, HIMRISS was significantly correlated with ASDAS-ESR, ASDAS-CRP, BASDAI, ESR, and CRP levels (all p < 0.05) in the TNF-α group at week 52, but the correlations were not significant at baseline. In addition, changed levels of HIMRISS between baseline and week 52 were significantly correlated with ASDAS-ESR changes in the TNF-α group. In the control group, HIMRISS was not significantly correlated with clinical outcomes at baseline and week 52, as well as changed levels (**Supplementary Table S2**).

## Discussion

This study demonstrated that treatment with TNF-α inhibitors could achieve significant clinical remission in patients and can manifest changes on MRI for hip arthritis in patients with SpA. In the TNF-α inhibitor group, disease activity indexes including BASDAI, ASDAS, ASAS remission rate, and hip function demonstrated superior improvement as compared with the control group. Additionally, the HIMRISS method could detect obviously better improvement in hip inflammation, based on overall MRI slices, under TNF-α inhibitor treatment versus control treatment. TNF-α inhibitor therapy was confirmed to be a better treatment option for patients with SpA and hip involvement.

Previous studies of therapy in SpA have mainly focused on axial spine radiology, and, to some extent, peripheral arthritis or enthesitis (5, 21). Hip involvement is difficult to cure, and it is considered an important cause of disability and poor prognosis in SpA, especially in ankylosing spondylitis (AS) (17, 22). There has been greater attention recently regarding hip arthritis in SpA, and various clinical studies on TNF-α inhibitor treatment and classical disease-modifying anti-rheumatic drug (DMARD) treatment have been conducted to assess the clinical and/or radiographic progress of hip joint and function (23–25). TNF-α inhibitor treatment has been demonstrated to be effective in patients with SpA and hip involvement. However, the specific effect differences between TNF-α inhibitors and other agents have rarely been evaluated in long-term follow-up studies.

Among therapeutic studies of SpA patients with hip involvement, the BASRI-h method is often adopted to assess radiological changes of hip lesions (24). However, most hip involvement in AS is related to early coxitis and is characterized by inflammation of the subchondral BME and synovitis (7, 26). The BASRI-h method is performed based on structural damage, which may not sensitively reflect the treatment effect in patients during early and acute inflammation stages in short-time follow-up. Comparatively, MRI is considered to be a more sensitive assessment tool for hip inflammation and is especially helpful in early diagnosis (8, 23, 27).

Recent studies have shown that synovial enhancement with hyperintense signals on contrast-enhanced T1-weighted images is the most frequent imaging finding in early-stage hip involvement in AS (14). Joint effusion of the femoral neck and subchondral BME are frequent findings with hyperintense signals on T2-weighted images or STIR images (8, 27). However, lesion frequency analysis or description of characteristics cannot quantitatively reflect MRI changes under treatment. The HIMRISS can be used to systematically quantify inflammatory changes including BME of the femoral head, acetabular BME, and synovitis effusion of the hip based on overall MRI slices (10). The HIMRISS method has been previously applied in patients with SpA diagnosed with hip arthritis, and there is a significant correlation between MRI manifestations and Harris score or disease activity (11).

In past clinical studies, clinical manifestations assessed in patients with SpA and hip involvement mostly refer to global outcome indexes, including disease activity scores and inflammatory markers. Despite the fact that the association between hip impairment and axial disease in inflammatory back pain is still controversial (22), further evidence points to the fact that hip disease more frequently occurs in patients with SpA and severe axial disease (28). However, the treatment efficacy and changes in hip involvement during the early stage and long-term follow-up are still not fully understood. Thus, more detailed assessments of clinical and MRI manifestations are needed.

In this study, the clinical efficacy of TNF-α inhibitors was compared with that of control agents in a 1-year follow-up period. TNF-α inhibitors were demonstrated to be significantly more effective in improving disease activity and inflammation than usual DMARD treatment. All the clinical indexes of the TNF-α group including ESR, CRP, BASDAI, and ASDAS-CRP/ASDAS-ESR were significantly ameliorated at different follow-up times compared with baseline; this result is consistent with previous studies. Harris score was also significantly increased, which reflected outstanding improvement of hip function.

In this study, the HIMRISS method was used to quantify the acute and chronic inflammatory changes of hip arthritis in SpA under treatment with TNF-α inhibitors or control treatment. The results of the HIMRISS showed significantly better improvement on MRI in the TNF-α group than the control group at week 52. All HIMRISS parameters of the TNF-α group including BME of the femoral head, BME of the acetabulum, synovitis effusion, and total HIMRISS scores showed significant improvement, with p-values <0.001. These results indicated that, to some extent, TNF-α inhibitors could simultaneously achieve significant long-term remission on MRI and clinical remission versus control treatment, which showed no notable MRI remission. Thus, TNF-α inhibitors demonstrated outstanding advantages for patients with SpA and hip involvement.

We performed additional subgroup analyses to further compare the clinical effect among different TNF-α inhibitors. A previous study indicated that etanercept and adalimumab treatment led to similar ASDAS-CRP improvement in patients with AS (16). In this study, the ASAS remission rates and HIMRISS were simultaneously compared between etanercept/infliximab and adalimumab at follow-up time points. Results of the Kaplan–Meier test indicated that the difference in remission rates, including those of ASAS20, ASAS40, and ASAS50, and the ASAS partial remission rate were not significant among the different TNF-α inhibitors. At the same time, HIMRISS also had a similar improvement at week 52 between different TNF-α inhibitors with all p-values >0.5. This result indicated that all TNF-α inhibitors had a similar positive effect in patients with SpA complicated with hip involvement.

The follow-up cycle of hip joint function and clinical characteristics varies from 12 weeks to 24 months. In this study, we observed the treatment response between 12 and 52 weeks. Lian et al. and Huang et al. (23) demonstrated that TNF-α inhibitors could maintain stable hip function according to the Harris and clinical remission of hip arthritis in patients with SpA for 6 months. In this study, we simultaneously compared the effectiveness of TNF-α inhibitors and control therapy for the clinical outcome of hip arthritis in SpA from baseline to 52 weeks. Our results further confirmed that clinical remission levels at week 52 in the TNF-α inhibitor group were significantly better than those of the SSZ group in all clinical parameters including ESR, CRP, Harris, BASDAI, and ASDAS. TNF-α inhibitor treatment yielded rapid clinical remission within 12 weeks and hip function improvement, and this improvement tended to remain through week 52.

The correlation analysis between HIMRISS and systematic clinical symptoms only indicates significant correlations in TNF-α inhibitor group at week 52, but the correlations were not significant at other time points and not significant in the control group. This result indicated that clinical remission and imaging remission may not be achieved at the same time, and treatment effect needs to be evaluated by multi-aspects.

Based on the fact that the cost of TNF-α inhibitors is relatively high in comparison with that of classical DMARDs, the present results indicate a real-world characteristic at baseline that patients in the TNF-α group had more serious disease activity and shorter disease duration than those in the control group, which was consistent with previous studies (29). Patients with SpA who had active hip arthritis are recommended to receive TNF-α inhibitor therapy for quick pain reduction and symptom remission, based on the global effectiveness of TNF-α inhibitors (30). Although disease duration assessed by months at baseline is significantly longer in the control group than the TNF-α group, the difference of average disease duration between the two groups was less than 2 years. In addition, the systematic clinical indexes of ESR, CRP, B27 Harris, BASDAI, and ASDAS-CRP at baseline were not significantly different between two groups. To some extent, the disease activity is a little worse in the TNF group instead. The results showed that as a chronic disease, although the average duration of disease difference in SpA between the two groups was less than 2 years, it should not have a great impact on the treatment response in this study. Similarly, the baseline results of MRI analysis did not fulfill the criteria of no difference between the two groups, particularly regarding the parameters of acetabular BME score, total BME score, and effusion synovitis. The present real-world data revealed that both disease activity and MRI features in patients under treatment with TNF-α inhibitors are more serious. TNF-α inhibitor therapy is suggested in these patients to quickly improve clinical outcomes, relieve pain, and reduce adverse reactions. As a consequence of these real-world characteristics, the present results must be interpreted with caution.

Some limitations of this study should be acknowledged. First, the 1-year treatment and observation period were short; longer follow-up is needed. Next, this was a single-center study in real world, and population bias may influence the applications regarding treatment efficiency. Moreover, although the HIMRISS method was used based on accurate MRI slices, it is not commonly used; additional application and confirmation of this method are required in the future.

## Conclusion

The results of our quantitative assessment demonstrated that TNF-α inhibitors could significantly and simultaneously improve clinical outcomes and the treatment response on MRI for hip arthritis in patients with SpA. This improvement was achieved quickly, within a 12-week treatment period, and was maintained in long-term follow-up. Our findings support a superior curative effect of TNF-α inhibitors over non-TNF-α inhibitor agents.

## Data Availability Statement

The original contributions presented in the study are included in the article/**Supplementary Material**. Further inquiries can be directed to the corresponding author.

## Ethics Statement

The studies involving human participants were reviewed and approved by the ethics committee of Xijing Hospital of China (instruction number: 20110303-7). The patients/participants provided their written informed consent to participate in this study.

## Author Contributions

KZ, YZ, and PZ contributed to the completion of the first draft and final version of the manuscript. PZ, KZ, and ZZ contributed to the initial design of the work. KZ, JD, ZZ, JJ, and PZ contributed to the clinical validation of the follow-up data. YL and MZ contributed to the interpretation of the MRI data. YZ, QH, WW, YW, and BZ contributed to the data acquisition and analysis. All authors contributed to the article and approved the submitted version.

## Funding

This work was supported by the National Basic Research Program of China (No. 2015CB553704) and the National Natural Science Foundation of China (No. 81871273).

## Conflict of Interest

The authors declare that the research was conducted in the absence of any commercial or financial relationships that could be construed as a potential conflict of interest.

## Publisher’s Note

All claims expressed in this article are solely those of the authors and do not necessarily represent those of their affiliated organizations, or those of the publisher, the editors and the reviewers. Any product that may be evaluated in this article, or claim that may be made by its manufacturer, is not guaranteed or endorsed by the publisher.

## Abbreviations

SpA, spondyloarthritis; BME, bone marrow edema; HIMRISS, Hip Inflammation MRI Scoring System; ESR, erythrocyte sedimentation rate; CRP, C-reactive protein; BASDAI, Bath Ankylosing Spondylitis Disease Activity Index; ASDAS, Ankylosing Spondylitis Disease Activity Score; ASAS, Assessment of SpondyloArthritis international Society; STIR, short tau inversion recovery; DMARDs, disease-modifying anti-rheumatic drugs; MTX, methotrexate; SSZ, sulfasalazine; HLA-B27, human leukocyte antigen B27; BASRI-h, Bath Ankylosing Spondylitis Radiology Index-hip score.
