# Supplementary material for: The Clinical and MRI Effect of TNF-α Inhibitors in Spondyloarthritis Patients With Hip Involvement: A Real-World Observational Clinical Study
Source: Front Immunol. 2021 Sep 29;12:740980. doi: 10.3389/fimmu.2021.740980 (PMC8511713; doi:10.3389/fimmu.2021.740980)
Supplement: Supplementary file 1 [file DataSheet_1.docx]

**Supplemental file.**

|  | **Group** | **Baseline** | **Week 52** | **Changes** | **Baseline vs.Week 52**  **P-value** |
| --- | --- | --- | --- | --- | --- |
| BME of femoral headmean  (0-65) | ETA | 11.26±7.08 | 8.53±5.94 | 2.74±0.48 | 0 |
|  | ADA+INF | 9.25±6.10 | 6.13±5.09 | 3.08±0.61 | 0 |
|  | **ETA vs. ADA+INF**  **P-value** | 0.899 | 0.527 | 0.763 |  |
| BME of acetabularmean  (0-35) | ETA | 7.72±3.90 | 6.14±4.08 | 1.58±0.30 | 0 |
|  | ADA+INF | 5.91±2.96 | 4.29±2.88 | 1.62±0.37 | 0 |
|  | **ETA vs. ADA+INF**  **P-value** | 0.014 | 0.06 | 0.948 |  |
| Total BMEmean  (0-100) | ETA | 18.98±10.02 | 14.67±9.34 | 4.32±0.69 | 0 |
|  | ADA+INF | 15.16±7.56 | 10.42±6.72 | 4.71±0.75 | 0 |
|  | **ETA vs. ADA+INF**  **P-value** | 0.041 | 0.058 | 0.370 |  |
| synovitis-effusion scoremean  (0-30) | ETA | 8.82±3.43 | 6.92±3.18 | 1.92±0.32 | 0 |
|  | ADA+INF | 9.04±3.18 | 8.08±2.39 | 0.96±0.31 | 0 |
|  | **ETA vs. ADA+INF**  **P-value** | 0.126 | 0.006 | 0.053 |  |
| Total HIMRISS ave  (0-130) | ETA | 27.80±11.00 | 21.58±10.36 | 6.24±0.76 | 0 |
|  | ADA+INF | 24.20±8.77 | 18.49±6.84 | 5.67±0.92 | 0 |
|  | **ETA vs. ADA+INF**  **P-value** | 0.224 | 0.023 | 0.847 |  |
| Total HIMRISS max  (0-130) | ETA | 31.32±12.06 | 24.04±11.03 | 7.33±0.84 | 0 |
|  | ADA+INF | 27.54±10.73 | 20.80±8.32 | 6.68±1.03 | 0 |
|  | **ETA vs. ADA+INF**  **P-value** | 0.556 | 0.072 | 0.834 |  |

**Table S1**. Sub-analysis of the HIMRISS scores from baseline to 52 weeks between two biological groups.Note: mean: the mean score of two hips; max: the max score of two hips.

Note: ave: the average score of two hips; max: the max score of two hips; ETA: etanercept; ADA: adalimumab; INF: infliximab; Changes: changed HIMRISS levels between baseline and week 52.

**Table S2**. The correlation analysis between HIMRISS and systematic clinical symptoms.

|  | **HIMRISS_**  **correlation** | **ASDAS-ESR** | **ASDAS-CRP** | **BASDAI** | **Harris** | **ESR** | **CRP** |
| --- | --- | --- | --- | --- | --- | --- | --- |
| **TNFα** | Baseline | 0.123 | 0.05 | 0.06 | -0.1 | 0.05 | 0.064 |
|  | 52W | 0.19** | 0.281** | 0.152* | -0.089 | 0.156* | 0.174* |
|  | Changes | 0.191* | 0.077 | 0.067 | 0.055 | 0.008 | 0.125 |
| **Non-TNFα** | Baseline | 0.045 | 0.101 | 0.235* | 0.216 | 0.039 | 0.087 |
|  | 52W | 0.041 | 0.002 | 0.002 | -0.007 | 0.04 | 0.082 |
|  | Changes | 0.113 | 0.157 | 0.13 | 0.041 | 0.115 | 0.048 |

Note: Changes were changed HIMRISS levels between baseline and week 52. The correlation coefficient was calculated between HIMRISS change level and clinical index change level. *: p<0.05; **: p<0.01.
